# Maternal milk and fecal microbes guide the spatiotemporal development of mucosa-associated microbiota and barrier function in the porcine neonatal gut

**DOI:** 10.1186/s12915-019-0729-2

**Published:** 2019-12-18

**Authors:** Hongbin Liu, Xiangfang Zeng, Guolong Zhang, Chengli Hou, Ning Li, Haitao Yu, Lijun Shang, Xiaoya Zhang, Paolo Trevisi, Feiyun Yang, Zuohua Liu, Shiyan Qiao

**Affiliations:** 1 State Key Laboratory of Animal Nutrition and Beijing Key Laboratory of Bio-Feed Additives, China Agricultural University, Beijing, China; 2 Present Address: Shenzhen Institute of Synthetic Biology, Shenzhen Institutes of Advanced Technology, Chinese Academy of Sciences, Shenzhen, 518055 China; 3 Department of Animal and Food Sciences, Oklahoma State University, Stillwater, OK USA; 4 Institute of Food Science and Technology, Chinese Academy of Agricultural Sciences, Beijing, China; 5 Department of Agricultural and Food Science, University of Bologna, Bologna, Italy; 6 Chongqing Academy of Animal Science, Chongqing, China

**Keywords:** Mucosal microbiota, Spatiotemporal colonization, Early life, Maternal microbial transmission, Immunologic maturation, Microbiota-host interaction

## Abstract

**Background:**

The early-life microbiota exerts a profound and lifelong impact on host health. Longitudinal studies in humans have been informative but are mostly based on the analysis of fecal samples and cannot shed direct light on the early development of mucosa-associated intestinal microbiota and its impact on GI function. Using piglets as a model for human infants, we assess here the succession of mucosa-associated microbiota across the intestinal tract in the first 35 days after birth.

**Results:**

Although sharing a similar composition and predicted functional profile at birth, the mucosa-associated microbiome in the small intestine (jejunum and ileum) remained relatively stable, while that of the large intestine (cecum and colon) quickly expanded and diversified by day 35. Among detected microbial sources (milk, vagina, areolar skin, and feces of sows, farrowing crate, and incubator), maternal milk microbes were primarily responsible for the colonization of the small intestine, contributing approximately 90% bacteria throughout the first 35 days of the neonatal life. Although maternal milk microbes contributed greater than 90% bacteria to the large intestinal microbiota of neonates upon birth, their presence gradually diminished, and they were replaced by maternal fecal microbes by day 35. We found strong correlations between the relative abundance of specific mucosa-associated microbes, particularly those vertically transmitted from the mother, and the expression levels of multiple intestinal immune and barrier function genes in different segments of the intestinal tract.

**Conclusion:**

We revealed spatially specific trajectories of microbial colonization of the intestinal mucosa in the small and large intestines, which can be primarily attributed to the colonization by vertically transmitted maternal milk and intestinal microbes. Additionally, these maternal microbes may be involved in the establishment of intestinal immune and barrier functions in neonates. Our findings strengthen the notion that studying fecal samples alone is insufficient to fully understand the co-development of the intestinal microbiota and immune system and suggest the possibility of improving neonatal health through the manipulation of maternal microbiota.

## Background

In the gastrointestinal (GI) tract, the mucosal surface uniquely serves as a conduit between the host immune system and the external environment, orchestrating a variety of physiological processes such as nutrient absorption and immune development [1, 2]. In this context, an extremely dense and diverse bacterial community resides on the mucosal surface and plays a critical role in host physiology and health [3]. The proper establishment of the intestinal microbiota in early life is well known to facilitate immune maturation [4, 5]. Colonization of neonatal, but not adult, germ-free mice with conventional intestinal microbiota has a positive long-term influence on the subsequent development of host innate and adaptive immunity [6, 7]. Conversely, aberrant microbial colonization during infancy is associated with a number of childhood diseases [8–10] and increases disease risk in later life [11–13].

Succession and maturation of the infant GI microbiome have been extensively studied [7, 14–16]. To date, most of these studies are, however, based on the use of fecal samples. Given that the maturation of the intestinal immune system significantly depends on mucosa-associated microbes [17–19] and that substantial differences exist between mucosa-associated and fecal microbiota [20–24], it is critically important to study the development of intestinal mucosa-associated microbiota in early life.

Pigs, sharing many common features in the gastrointestinal physiology, microbiology, genetics, and diet with humans, are an excellent animal model and have been widely used in biomedical research [25, 26]. In particular, in contrast to rodents, the sow-piglet dyad has been suggested as a more promising model for the human mother-infant dyad to study the development of the GI tract functions [26–29]. Recent studies have also shown the metagenomic profile and inter-individual variability to be more similar between pigs and humans than between mice and humans, making pigs a superior model for human GI microbiota research [30, 31]. Furthermore, confounding variables that are impractical to control in human studies could be controlled or avoided in swine experimentation. Thus, neonatal piglets are well suitable for in-depth studies of the initial colonization and development of infant intestinal mucosal microbiome.

The influence of the birth mode [32, 33], antibiotics [32, 34, 35], and nutrition [8, 36] on the development of infant GI microbiota has been well studied. However, little is known about the impact and contribution of different microbial sources from the mother and the environment, although existing evidence suggests that neonatal microbes are likely to come from the mother and immediate rearing environment [15, 37]. Recent studies suggested that the vagina, milk, and areolar skin of a mother contain diverse bacterial communities and are important sources of infant GI microbiota [38]. The neonatal environment also exerts a sustained influence on the development of infant intestinal microbiota [37, 39, 40]. However, no longitudinal studies have been conducted to analyze the relative contributions of these microbial sources to the colonization of neonatal GI.

In this study, we comprehensively surveyed the spatial and temporal development of mucosa-associated bacterial community and its correlation with the host gene expression along the GI tract in healthy neonatal piglets. In addition, relative contributions of different microbial sources from the mother (vagina, areolar skin, feces, and milk of the sow) and the neonatal environment (farrowing crate and incubator) were also assessed.

## Results

### Structure of the mucosa-associated microbiota is relatively stable in the small, but not the large, intestine in early life

After quality filtering and assembly, 13,768,547 16S rRNA gene sequences were obtained from 367 mucosal bacterial DNA samples of Landrace and Rongchang piglets from birth to 35 days (average of 37,516 sequences/sample, Additional file 1: Figure S1). To avoid biases generated by the differences in sequencing depth, we rarefied each sample to a depth of 27,848 sequences/sample prior to performing the following analyses. Rarefaction curves of Chao1 and Shannon diversity indices calculated at the OTU level (Additional file 2: Figure S2) reached a plateau, suggesting that the majority of microbial diversity had been sufficiently captured.

**Fig. 1:**
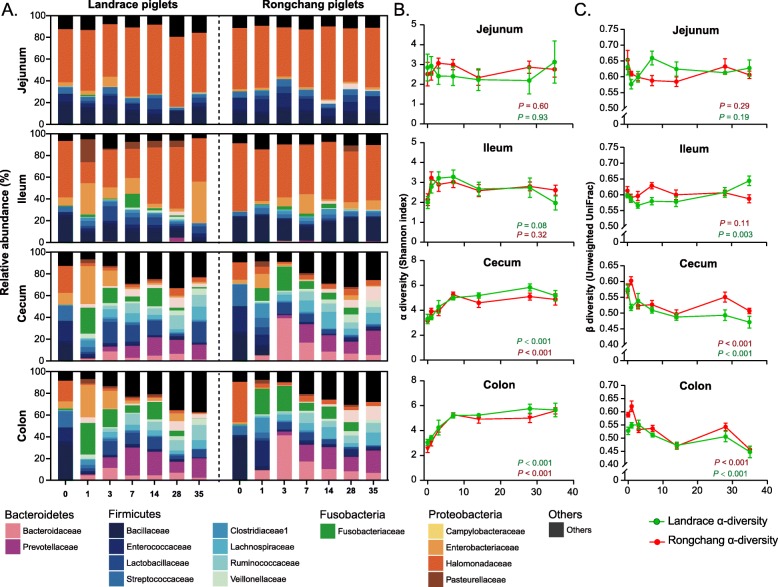
Development of the mucosa-associated microbiota in the small and large intestines of Rongchang and Landrace piglets during the first 35 days after birth. **a** Stacked area plot displaying the changes in the relative abundance (%) of the 15 most abundant bacterial families with age. **b** The shift of α-diversity (Shannon index) with age across 4 intestinal segments. **c** The shift of β-diversity (unweighted UniFrac distance) with age across 4 intestinal segments. The β-diversity at each time point is the average distance of one sample to all other samples at that time point

Fecal microbiota is known to gradually increase in the richness and diversity with age [41, 42]. In pigs, mucosa-associated microbiota in the small intestine (jejunum and ileum) of piglets was dominated by *Halomonadaceae*, whereas that in the large intestine (cecum and colon) was much more diverse with no obvious dominant bacterial taxa in the first 35 days of life (Fig. 1a). Overall, the small intestine harbored mostly Proteobacteria (*Halomonadaceae* and *Enterobacteriaceae*) and a small fraction of Firmicutes (*Bacillaceae*, *Enterococcaceae*, and *Streptococcaceae*), while the large intestine consisted of multiple families of Firmicutes, Bacteroidetes, Fusobacteria, and much reduced abundance of Proteobacteria. Moreover, we observed distinct trajectories of bacterial succession and maturation in different intestinal segments for both breeds. As expected, the microbiota experienced a dramatic shift in both the small and large intestines on the first day after birth. To our surprise, the microbiota in the small intestine quickly became stabilized from day 3 to day 35, while those in the large intestine (cecum and colon) failed to achieve the equilibrium until after 7–14 days (Fig. 1a). An abrupt disappearance of *Bacillaceae* and *Enterococcaceae* was observed in the cecum and colon shortly after birth, followed by a gradual increase in *Lactobacillaceae*, *Lachnospiraceae*, *Ruminococcaceae*, and *Veillonellaceae* as piglets aged.

PERMANOVA analyses revealed that mucosa-associated intestinal microbiome was primarily shaped by biogeographic location (*R*^2^ = 0.357, *P* < 0.001, weighted UniFrac, Table 1). Although the breed had a significant effect (*P* < 0.001) on the GI microbiota structure as measured by unweighted UniFrac and Bray-Curtis metrics, but not weighted UniFrac (*P* = 0.064, Table 1), extremely small *R*^2^ values (ranging between 0.011 and 0.012) indicated that the breed effect is negligible. Indeed, similar results were observed between two breeds at birth as well (Additional file 3: Figure S3A), suggesting that breed had little influence on the phylogenetic composition of the mucosa-associated microbiome. Therefore, samples between the two breeds within each time point and intestinal segment were grouped together in subsequent analyses.

**Table 1:** Factors contributing to the variation in intestinal mucosa-associated microbiota

Items	Weighted UniFrac		Unweighted UniFrac		Bray-Curtis	
	*R*^2^	*P* value*	*R*^2^	*P* value*	*R*^2^	*P* value*
Intestinal segment	0.357	*< 0.001*	0.108	*< 0.001*	0.229	*< 0.001*
Age	0.136	*< 0.001*	0.110	*< 0.001*	0.123	*< 0.001*
Weaning	0.019	*< 0.001*	0.032	*< 0.001*	0.021	*< 0.001*
Breed	0.006	0.064	0.011	*< 0.001*	0.012	*< 0.001*
Sex	0.002	0.525	0.004	0.061	0.003	0.315

*PERMANOVA was performed, and *P* values in italics represent statistical significance (*P* < 0.05)

**Fig. 2:**
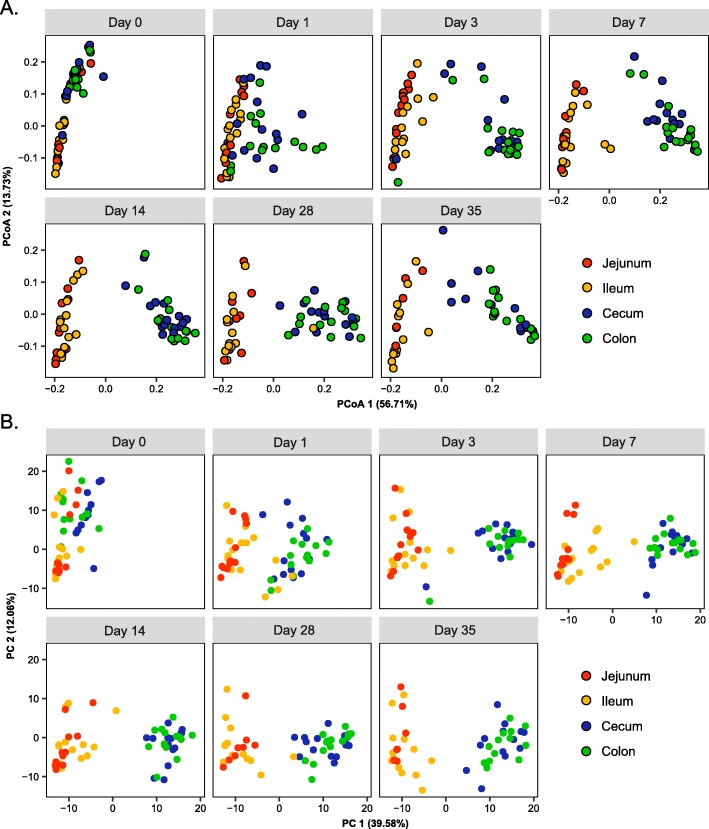
β-diversity of the mucosa-associated microbiota across different intestine locations. **a** PCoA of phylogenetic community composition based on weighted UniFrac distance. Population-level PERMANOVA statistics are detailed in Table 1. **b** Principal components analysis (PCA) of predicted functional genes of mucosa-associated microbiota at KEGG level 3

Consistently, no obvious changes in the α-diversity of mucosa-associated microbiota occurred in the small intestine across different ages, while the α-diversity of the large intestinal microbiota was significantly increased in the first 7–14 days before becoming stabilized (*P* < 0.001, Fig. 1b), except for a transient decline observed on day 1 (Additional file 3: Figure S3B). The β-diversity analysis (unweighted UniFrac) showed a similar trend. The mucosa-associated microbiota remained relatively stable in the small intestine, while diverged from the day 0 microbiota rapidly in the large intestine in the first week before becoming more or less stabilized (Fig. 1c). To further reveal the mature pattern of mucosa-associated microbiota in the small and large intestines, PCoA was performed based on the phylogenetic metrics (weighted and unweighted Unifrac, Fig. 2a, Additional file 4: Figure S4A) or taxonomic metric (Bray-Curtis, Additional file 4: Figure S4B). Nearly identical patterns were yielded from three metrics. While the small intestinal microbiota of different ages were clustered largely together, the large intestine was similar in the microbiota composition to the small intestine at birth, but became progressively divergent with age, resulting in two rather distinct microbial communities between the small and large intestines by day 35 (Fig. 2a; Additional file 4: Figure S4, Table 2). Together, these observations suggested a rather different succession pattern of mucosa-associated microbiota between the small and large intestines.

**Table 2:** Intestinal location-dependent influence of age on the mucosa-associated microbial community

Items	Weighted UniFrac		Unweighted UniFrac		Bray-Curtis	
	*R*^2^	*P* value	*R*^2^	*P* value	*R*^2^	*P* value
Jejunum	0.086	0.404	0.089	0.167	0.084	0.392
Ileum	0.076	0.208	0.094	*< 0.001*	0.109	*0.005*
Cecum	0.426	*< 0.001*	0.286	*< 0.001*	0.32	*< 0.001*
Colon	0.463	*< 0.001*	0.312	*< 0.001*	0.339	*< 0.001*

PERMANOVA was performed to test the effect of days after birth on mucosa-associated microbiota in different intestinal segments. *P* values in italics represent statistical significance (*P* < 0.05)

### Mucosa-associated microbiota in the large intestine is predicted to be progressively divergent in functions from that in the small intestine

The difference in the composition of mucosa-associated microbiota between the small and large intestines suggested a distinct functional maturation process of the microbiome. To investigate how the functional profiles of the mucosa-associated microbiome change during early life, PICRUSt analysis [43] was performed. Similar to our earlier observations on the bacterial community structure, the microbiota function was predicted to be similar between the small and large intestines at birth (Fig. 2b). While the bacterial function was relatively stable in the small intestine across the ages, the large intestinal microbiota became progressively divergent as the animals aged (Fig. 2b). In the large intestine, 259 functional pathways were identified to be significantly altered along with piglet development. Specifically, the most highly enriched pathways were predicted to be involved in genetic information processing such as regulation of transcriptional factors, nucleotide excision repair, lysine biosynthesis, homologous recombination, and mismatch repair (Additional file 9: Table S2). In contrast, only 4 pathways were significantly altered (*P* < 0.05) during small intestinal development, although approximately an equal number of functional pathways were identified in both (small intestine, *N* = 263; large intestine, *N* = 264). Moreover, out of 261 most prevalent KEGG pathways that were present in at least 50% samples, 241 were significantly different between the small and large intestines (*P* < 0.05, Additional file 10: Table S3). In particular, the small intestinal microbiome was associated with enrichment of the pathways in biodegradation and metabolism of xenobiotics, whereas the pathways involved in glycan biosynthesis and metabolism, replication and repair, energy metabolism, translation, and carbohydrate metabolism were more abundant in the large intestine. Together, these data suggested that, in addition to the structural differences, functional profiles of the bacterial community are also vastly different between the small and large intestines. While it was relatively stable in the small intestine, the microbiota function shifted towards an adult-like configuration in the large intestine.

### Maternal milk and fecal microbiota are the major contributors of neonatal intestinal mucosa

**Fig. 3:**
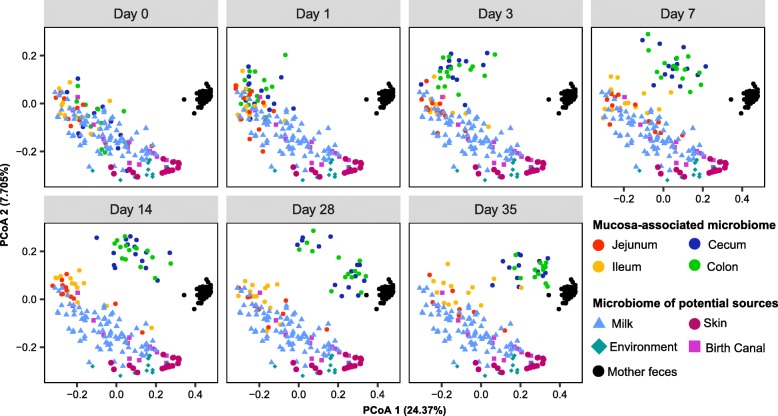
β-diversity of the mucosa-associated microbiota, milk, skin, vagina and feces of sows, and environmental microbiota. PCoA of phylogenetic community composition based on unweighted UniFrac distance

To analyze the potential sources of the microbial assembly in infant piglet intestinal mucosa, fecal and milk samples as well as the areolar skin and vaginal swabs were collected from sows. Farrowing crates and incubators were also swabbed as environmental samples. PCoA using unweighted UniFrac distance showed close clustering of the milk and small intestine samples, while the large intestinal microbiota was clustered with the small intestine at birth, but gradually diverged with age, resembling more and more the fecal microbiota of sows (Fig. 3). PCoA using weighted Unifrac and the Bray-Curtis metrics showed similar clustering patterns (Additional file 5: Figure S5), implying that the maternal milk and fecal microbiome might serve as microbial reservoirs for vertical transmission.

**Fig. 4:**
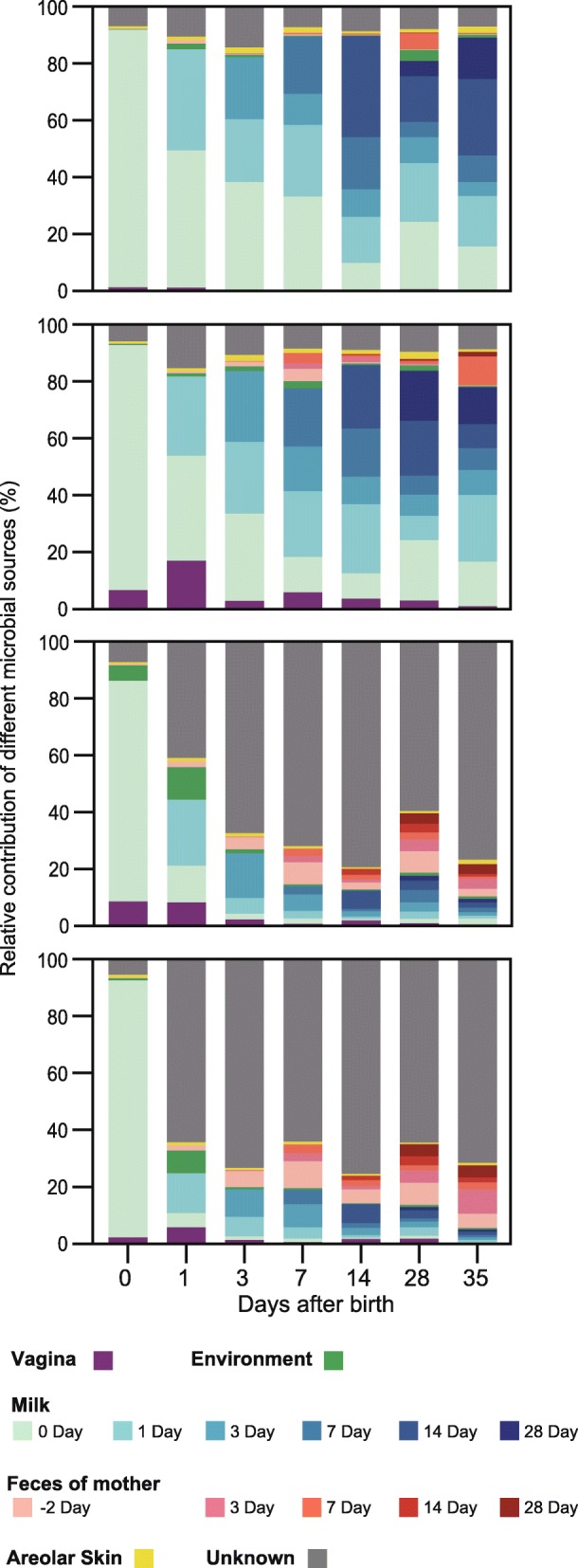
Dynamic contributions of different microbial sources to the neonatal gut mucosal microbiota during the first 35 days. The proportion of microbiota from the jejunum, ileum, cecum, and colon samples of piglets are estimated to originate from different maternal and environmental sources (colored regions), using bacterial source-tracking

SourceTracker [44] was performed to further analyze the relative contributions of different microbial sources from the mother and birth environment on the initial colonization of neonatal GI. Obviously, maternal milk was the primary contributor of the microbiota in the small intestine, accounting for approximately 90% of the microbiota throughout the first 35 days, even after the introduction of solid creep feed on day 7 (Fig. 4). In contrast, although more than 90% of the large intestinal microbiota of piglets also originated from maternal milk, its contribution gradually declined with age (Fig. 4). Milk microbes contributed approximately 20% of the microbiota in the large intestine on day 3, which was further diminished to less than 5% in the colon and 10% in the cecum. On the other hand, the contribution of maternal fecal microbiota became progressively more prominent with age. Albeit with virtually no contributions at birth, fecal microbes of sows contributed 7–20% of the microbiota in piglet’s large intestine between days 7 and 35 (Fig. 4). Similarly, pairwise β-diversity comparisons showed that milk microbiota closely resembled that of the small intestine initially, while fecal microbiota became progressively similar to the large intestinal microbiota (Additional file 6: Figure S6). Maternal vaginal microbiota contributed 6–16% of mucosa-associated microbiota in the ileum, cecum, and colon on day 1; however, such an effect occurred only transiently and quickly diminished by day 35 (Fig. 4). Similarly, the neonatal birth environment contributed 2–10% of mucosal microbiota in the large intestine within the first 2 weeks, and its contribution further diminished with age (Fig. 4).

**Fig. 5:**
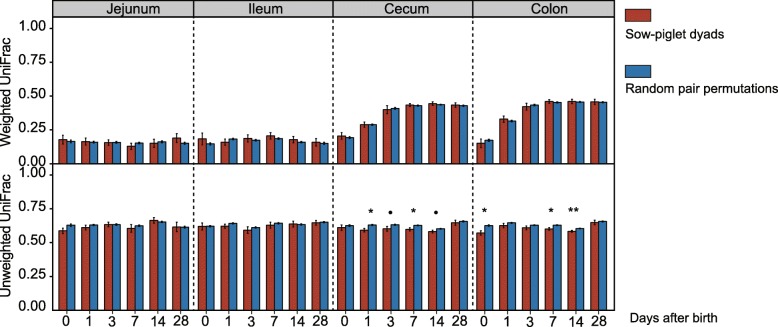
Distance comparison of microbial communities between true sow-piglet dyads and random pairs for milk microbiota (values are means ± SE; significance between the intestinal segments was determined by pairwise Kruskal-Wallis test; *P < 0.05; **P < 0.01; ***P < 0.001)

To further confirm bacterial transmission from sows or environment to infant piglets, we hypothesized that a piglet’s intestinal mucosa-associated microbiota more resembles its mother’s microbiota than a random sow’s. A closer resemblance in unweighted Unifrac distance between sow milk microbiota and piglet’s microbiota in the large intestine was observed among sow-piglet dyads than random pairs (*P* < 0.05; Fig. 5). Similar results were also found between the vaginal and environmental microbiota and piglet’s large intestinal microbiota (*P* < 0.05; Additional file 7: Figure S7), further corroborating the occurrence of microbial transmission.

Next, we sought to identify specific OTUs transmitted from sow and birth environment to piglets. A total of 24 OTUs were significantly shared among sow-piglet dyads as compared to random pairs and thus identified as transmitted bacterial taxa (Additional file 11: Table S4). Of the 24 OTUs, 16 belonged to Firmicutes and 5 belonged to Proteobacteria. Genus-level annotation of these OTUs revealed that *Corynebacterium*, *Bacillus*, *Lactococcus*, *Staphylococcus*, *Romboutsia*, *Ruminococcaceae TCG-005*, *Escherichia-Shigella*, *Streptococcus*, and *Halomonas* were likely transferred from the maternal and birth environment to the piglet’s intestine (Additional file 11: Table S4). A species (OTU468) of *Christensenellaceae*, the most heritable taxon previously reported [45], was likely to be transferred from the feces of sows to the large intestine of piglets. Interestingly, half of these bacterial transmissions (19/38) occurred through milk and the vagina within the first 3 days, while the other half occurred through maternal feces and environment in the following several weeks. More importantly, most of these bacterial transmissions (36/38) occurred in the ileum, cecum, and colon (Additional file 11: Table S4). Although the underlying mechanism remains unclear, this intestinal segment-specific transmission is likely due to the difference in the ability of microbes to colonize different ecological niches in the GI [46, 47].

### Maternally transmitted microbes are involved in the regional expression of intestinal immune and functional genes

To examine whether mucosa-associated bacteria are associated with functional development of the neonatal GI, nine genes known to be involved in immune and barrier functions were selected and quantified in the jejunum, ileum, and colon samples at different ages. Among them, porcine β-defensin 1 (PBD1), PBD2, and regenerative III protein (RegIII) are the major host defense peptides against infections [48, 49], while mucin (MUC) 1, MUC2, and MUC13 are the important members of the mucin family forming the protective mucus layer along the intestine [50]. Aryl hydrocarbon receptor (AHR) and Toll-like receptor 4 (TLR4) are important receptors mediating host inflammatory and immune response [51, 52], and interleukin (IL)-10 is a well-known anti-inflammatory cytokine [53].

**Fig. 6:**
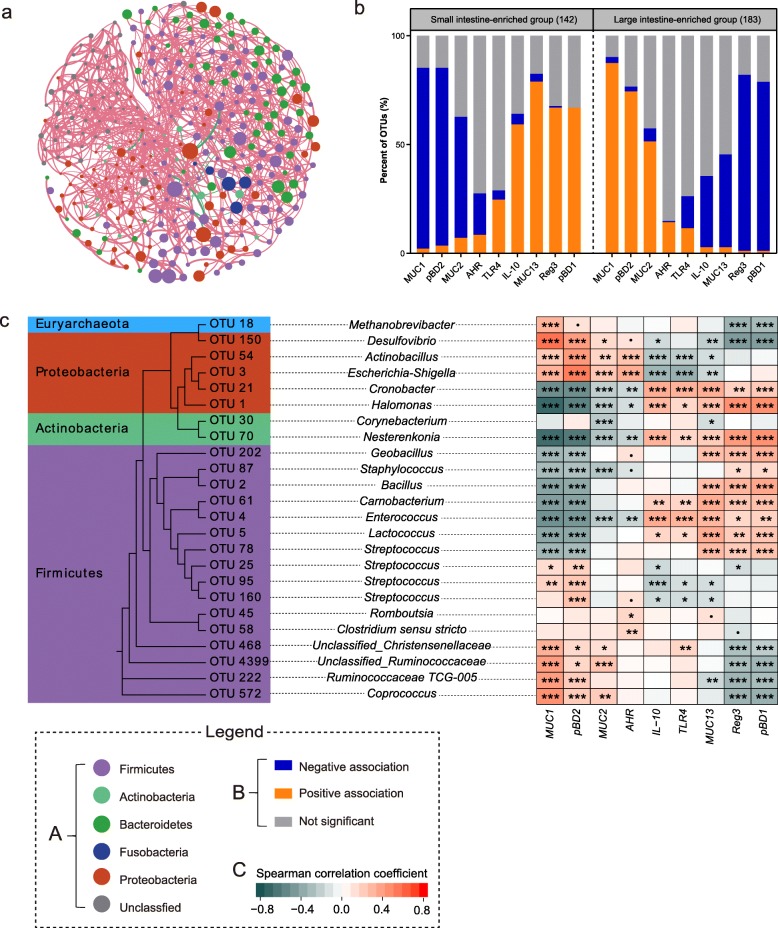
Involvement of mucosa-associated microbiota in the spatial expression of intestinal functional genes. **a** Network plots of OTUs (represented by nodes) that are significantly associated with the expression of intestinal genes. Significant correlative associations between OTUs were determined based on the SPIEC-EASI pipeline. Edge color represents positive (pink) and negative (green) correlations, and the edge thickness is equivalent to the magnitude of the correlation coefficient. SPIEC-EASI correlations with a magnitude of < 0.05 were not shown. The diameter of each node is proportional to the average abundance of each OTU across all samples, while each filled color corresponds to a bacterial phylum. **b** Spatial correlation pattern between the small and large intestine-enriched OTUs with the expression level of the intestinal function genes. The full list of significant correlative associations is presented in Additional file 14: Table S7. **c** A list of bacterial OTUs that are transmitted from sow and birth environment to the piglets showing a significant correlation with the expression of intestinal functional genes. The left panel is the phylogenetic tree of transmitted OTUs, whereas the right panel is the heat map of Spearman’s rank correlation coefficient between 24 transmitted OTUs and expression levels of intestinal genes (^•^*P* < 0.1, *P < 0.05, **P < 0.01, ***P < 0.001). The background color corresponds to the phyla to which the OTU belongs. Spearman’s rank correlation coefficient is indicated using a color gradient: red indicates positive correlation; cyan, negative correlation

As expected, most intestinal genes were differentially expressed in the jejunum, ileum, and colon of piglets (*P* < 0.05; Additional file 12: Table S5), consistent with the idea of regional specialization and maturation of the intestinal immune and barrier functions as animals age [54]. For instance, the expression level of *MUC2* was significantly elevated in the colon with age but remained relatively stable in the jejunum and ileum (Additional file 13: Table S6). In contrast, a steady increase in the expression of *TLR4* and *IL-10* was observed across three intestinal segments (*P* < 0.05; Additional file 12: Table S5). Furthermore, the expression of intestinal genes was significantly correlated with the relative abundance of a number of OTUs (*P* < 0.05; Additional file 14: Table S7). Surprisingly, most of these OTUs showed a positive correlation with each other (Fig. 6a), implying a possible existence of the microbial consortia that may promote mutual growth and guide the expression of intestinal immune and barrier function genes. Based on the differential abundance between small and large intestine, these OTUs were stratified into two groups, the small intestine-enriched group and the large intestine-enriched group (Additional file 14: Table S7). The small intestine-enriched OTUs were mostly associated with highly abundant Proteobacteria, but not Bacteroidetes (Additional file 14: Table S7). Intriguingly, an opposite correlation pattern was observed between the two groups and intestinal gene expressions. For instance, the small intestinal group showed a negative correlation with the expression of *MUC1*, which became positive for the large intestinal group (Fig. 6b; Additional file 14: Table S7). Nearly identical correlation patterns were observed between the two groups and the expressions of remaining genes (*P* < 0.05, Fig. 6b; Additional file 14: Table S7). Importantly, all vertically transmitted bacteria were significantly correlated with the expression levels of intestinal immune and barrier function genes (Fig. 6c), implying that maternally transmitted bacteria are critically involved in the maturation of immune and barrier functions in the neonatal GI.

## Discussion

The GI microbiota in early life has long-term implications in host physiology and health [6, 55]. Because the GI microbiota varies greatly along the intestinal tract and undergoes substantial changes with age, it is important to understand initial colonization and succession of microbiota in different segments of the neonatal GI. However, most studies on the development of infant microbiome were conducted only with stool samples due to technical and ethical limitations [14, 15, 56]. Here, using mucosa-associated microbiota from the small and large intestines of piglets as well as maternal and environmental microbiota, we systematically studied the development and origin of piglet’s GI microbiota and its impact on intestinal innate immune and barrier functions.

Fecal microbiota undergoes progressive succession before maturation [57]. Unexpectedly, we observed for the first time distinct succession patterns of the mucosa-associated microbiota between the small and large intestines. Despite a similar initial composition and predicted functional profile at birth, mucosal microbiota in the small intestine remains relatively stable, while that in the large intestine undergoes progressive expansion and diversification as soon as 24 h after birth and continuously shifts in the first 35 days of age. These temporal and spatial dynamics resemble the development of the lumen-associated microbiota, which showed similar initial structure across different intestinal segments (duodenum, jejunum, ileum, cecum, colon, and rectum) on postnatal day 1, but evolved and quickly differentiated at later intervals [58]. Similarly, the changes of the lumen-associated microbial community in the small intestine are negligible during the nursing (postnatal day 7 to day 35) and weaning periods (postnatal day 120 to day 180); in contrast, that in the large intestine undergoes considerable changes. However, we did not observe either similar composition or changes in the microbiota of the small intestine at the taxonomic level as previously reported [58]. Nevertheless, it should be noted that different intestinal sites, luminal digesta and mucosa, were sampled in two studies. Indeed, GI microbiota differs radically in their functional potential, density, and composition from the mucosa to the lumen [23, 59]. Second, it is likely that microbial compositions vary according to the genetic background of pig and many other factors [30]. Lastly, the small cohort (*N* = 5) and substantial inter-individual variation in the lumen-associated microbiota in the research conducted by Liu et al. [58] might also lead to the contrary observations. Collectively, these observations suggested a spatiotemporal developmental and maturation pattern of the mucosa-associated bacterial community.

In this study, we detected diverse microbiota in different segments of the intestine and different body sites at birth, consistent with earlier detection of bacteria within the first-pass meconium [60, 61], placenta [62], and amniotic fluid [63] from healthy term infants. The first meconium microbiota is thought to reflect the in utero environment since the fetus could swallow amniotic fluid in the womb. Indeed, the meconium has been shown to harbor a microbial community resembling those in the amniotic fluid and placenta [60, 63, 64]. Furthermore, maternal transmission of bacteria to the fetal GI during gestation has been observed in murine models [65], reinforcing the idea that microbial colonization of the fetus may occur before birth.

We have also revealed that mucosa-associated intestinal microbiota is primarily derived from vertical transmission of the microbes in maternal milk and the GI. Milk contributes greater than 90% of mucosa-associated microbiota in the small intestine of the neonates in the first 35 days after birth. The contribution of the milk microbes to the neonatal mucosal microbiota is gradually declined in the large intestine, but with a concomitant increase in the contribution of maternal fecal microbes. In agreement with our study, up to 30% of the infant fecal microbes were previously reported to come from milk and declined as the infant ages [38]. Perhaps not coincidently, the significance of the milk microbiota is underscored by the observation that the GI microbiota is drastically different between formula-fed and breastfed infants [32, 66]. A more recent study involving 25 mother-infant pairs concluded that maternal GI microbiome is also a major source of neonatal GI microbes [67].

We found that vaginal microbes of sows colonize neonatal GI mucosa at day 1, consistent with a number of earlier studies showing distinct GI microbiota between infants delivered vaginally and by cesarean section [15, 33]. However, this “vaginal seeding” is likely transient after birth. We observed that the contribution of vaginal microbiota became diminished quickly in a few days, which is in line with the results showing that the influence of birth mode failed to be detected in 6 weeks [15, 56, 67]. Indeed, we found that the transfer of maternal vaginal and milk microbes mostly occurred within 24 h shortly after birth, whereas that of maternal GI and environment microbes mostly occurred after 7 days. On the other hand, the initial acquisition from the mother could prepare the newborns for host-microbial symbiosis. Indeed, paralleling the frequent occurrence of the maternal bacterial seeding within 3 days after birth, a decrease at days 1 and 3 in microbial diversity has been observed. Similar observations have been made in mice and human infants [15, 68], suggesting a selective effect of the pioneer microbes from maternal milk and vagina on the infant GI microbiota colonization [69], while the higher bacterial diversity at birth could be explained by the in utero colonization of the neonates.

The infant’s intestinal microbiota is believed to guide the development and maturation of the intestinal immunity and barrier function [5, 70]. Aberrant mucosal defense and barrier integrity of germ-free mice can be rescued by transplantation with normal microbiota [71, 72]. In this study, we demonstrated that the relative abundances of a number of mucosa-associated microbes are significantly correlated with the expression of multiple intestinal genes known to be involved in innate immunity and barrier function. Importantly, these bacteria can be clustered into two groups enriched in either the small or large intestine, showing a strong correlation with the spatial expression pattern of the intestinal functional genes. In line with our observation, induction of TH17 cells requires the colonization of segmented filamentous bacteria (SFB) in the terminal ileum, which subsequently leads to increased expression of the genes associated with inflammation and antimicrobial defense [17]. Furthermore, microbes are known to differentially colonize along the intestinal mucosa surface [47, 73] and has a profound impact on local expressions of a multitude of host genes [74, 75]. Our findings have further supported the notion on the involvement of mucosa-associated microbiota in the site-specific development and maturation of intestinal mucosal immunity and barrier function.

Importantly, we have revealed that most maternally transmitted bacteria show a strong correlation with the expression of intestinal functional genes, suggesting a significant involvement of maternally derived microbes in the maturation of intestinal function. In agreement with this, human milk microbes have been shown to contribute to the immune development and maturation [76, 77], and cesarean increases the risk of allergic and autoimmune diseases in the offspring [78–80], while breastfeeding has been linked to the enhanced immunity and decreased risks for illnesses such as obesity [81, 82]. Our results have further supported the rationale for maternal bacterial seeding such as vaginal delivery and breastfeeding. Further studies on the roles of maternally derived microbes in infant health are warranted.

## Conclusion

We revealed for the first time that mucosa-associated microbiota in the small intestine (jejunum and ileum) remains relatively stable in early life, while those in the large intestine (cecum and colon) become quickly diversified in both the structure and function. Moreover, the neonatal GI microbiota is primarily shaped by the microbes from maternal milk and feces. We further demonstrated a strong temporal and spatial correlation between maternally derived microbiota and the expression pattern of host immune and functional genes along the intestinal mucosal surface, suggesting a potentially critical involvement of maternal microbiota in the site-specific development and maturation of intestinal mucosal immunity and barrier function. A better understanding of the succession of mucosa-associated intestinal microbiota throughout the neonatal GI may lead to new approaches to precise therapeutic manipulations.

## Materials and methods

### Experimental design and sample collection

Healthy, third-parity purebred Rongchang sows (*n* = 8) and Landrace sows (*n* = 8) with a similar expected delivery date were selected for this study. Each sow was individually housed in a different environmentally controlled room under standard management with access to a common non-medicated diet. To avoid cross-contamination, the building was decontaminated prior to the beginning of the trial and each breed was housed on a separate side of the building. After delivery, newborn piglets were co-housed with sows by litter and ear-notched for individual identification. Suckling piglets were offered a common creep feed ad libitum at day 7 and weaned at day 28. All piglets remained in nursing pens for another week till day 35, while sows were removed from the piglets at day 28.

One piglet from each litter was randomly selected and euthanized with Zoletil 50® (Virbac, Carros, France) at birth and at days 1, 3, 7, 14, 28, and 35 (Additional file 1: Figure S1), while ensuring half males and half females for each breed at each time point. Mucosa-associated microbiota was collected from a middle section of the jejunum, ileum, cecum, and colon of each piglet for bacterial DNA isolation as previously described [83] and adjacent intestinal segments were also collected for RNA extraction. A composite sample of milk was collected from each sow at the same time points after farrowing and continued until weaning. In addition, fresh fecal samples were taken from sows 2 days before farrowing and at days 3, 7, 14, and 28 after farrowing (Additional file 1: Figure S1). Additionally, vaginal and areolar skin swabs were taken from sows immediately after giving birth. A vaginal swab was taken by swirling a sterile cotton swab (Kangjie Medical Devices Co., Jiangsu, China) near the mid-vaginal canal six times, while areolar skin around the teat (approximate 10 cm in diameter) was swabbed with saline-lubricated sterile swabs. Farrowing crates and incubators were also sampled with sterile saline-soaked swabs. Each of these three specimens was sampled in triplicate for microbial analysis. All samples were immediately snap-frozen in liquid nitrogen and stored at − 80 °C for further analysis.

### Bacterial DNA extraction and 16S r RNA gene sequencing

Microbial DNA was extracted from the intestinal mucosa-associated microbiota, feces, and swabs using QIAamp DNA Stool Mini Kit (Qiagen, Duesseldorf, Germany). Microbial DNA was extracted from the milk using a DNeasy PowerFood Microbial Kit (Qiagen). Both procedures were carried out according to the manufacturer’s instructions, with an addition of a bead-beating step using 0.25 g of 0.15 mm garnet beads and 0.25 g of 0.1 mm zirconia beads. DNA was quantified with a NanoDrop 2000 spectrophotometer (Thermo Fisher Scientific, DE, USA), and the integrity was checked by 1% agarose gel electrophoresis. Amplification of the V3-V4 region of bacterial 16S rRNA genes was carried out as previously described [84]. Briefly, bar-coded universal primers 341F and 806R were designed for PCR amplification with initial denaturation at 95 °C for 5 min and 27 cycles of denaturation at 95 °C for 30 s, annealing at 55 °C for 30 s, and elongation at 72 °C for 45 s, followed by a final extension at 72 °C for 10 min. The PCR products were gel purified, quantified via NanoDrop™ 2000 spectrophotometer (Thermo Scientific), pooled at equal molar ratios, and sequenced on Illumina HiSeq 2500.

### Sequence analysis

Raw sequence data from a total of 556 samples were processed using QIIME (version 1.8.0). Sequences were de-noised using denoise_wrapper.py. High-quality sequences were clustered into distinct operational taxonomic units (OTUs) using UCLUST with a 97% threshold of the pairwise identity. Chimeric sequences were removed using identify_chimeric_seqs.py. The OTU table was filtered using filter_otus_from_otu_table.py. The most abundant sequence was picked for each OTU and taxonomically assigned using the SILVA reference database (Version 111) [85]. Representative OTUs were aligned using PyNAST [86] to build a phylogenetic tree with FastTree [87], which was used subsequently to estimate the α- and β-diversity. Microbial community distances were calculated using Bray-Curtis, weighted, and unweighted UniFrac distance metrics [88].

### Gene expression and qPCR

RNA isolation and quantification of intestinal segments were performed as previously described [89] using the primers listed in Additional file 8: Table S1. All reactions were run in triplicate. Relative gene expression was calculated according to the ΔΔCt method [90] using porcine β-actin as the reference gene.

### Data analysis and statistics

Statistical analysis was performed using SPSS 22.0 (SPSS, Chicago, IL, USA) and R programming. For normally distributed continuous variables, the mean values were examined using an unpaired Student’s *t* test or one-way ANOVA with Tukey’s post hoc test. The α-diversity was calculated using Chao1 and Shannon diversity indices and compared using Kruskal-Wallis tests with Benjamini-Hochberg correction. Principal coordinates analysis (PCoA) was performed on the Bray-Curtis, unweighted, and weighted UniFrac distance metrics to visualize the relationships between the samples. Permutational multivariate analysis of variance (PERMANOVA) using the adonis function in vegan with 9999 permutations was performed to analyze the distance metrics for factors that shape mucosa-associated microbiota.

PICRUSt analysis was applied to infer putative metagenomes from the 16S rRNA gene profiles [43]. Differences in the abundance of KEGG pathways between the groups were analyzed using STAMP software [91] and Welch’s *t* test with Benjamini-Hochberg correction. To estimate the sources of microbial communities observed at different intestinal segments and days after birth, we used SourceTracker (v1.0), a Bayesian approach for bacterial source tracking [44]. Samples collected from different maternal body sites and the birth environment were designated as sources, and samples from the intestinal mucosa of the piglets were tagged as sinks. SourceTracker R package (Version 1.0) was used to perform the analysis with default parameters. OTU sharing was defined as the percentage of mother-infant dyads in which a given OTU was found in both members. Permutation testing with randomly shuffled mother-infant pairings was used to assess the significance of OTU sharing as previously described [38]. Fisher’s exact test was employed to test the association between frequencies within sow-piglet dyads as described [92]. Because both tests ignore the OTUs that are present in all samples, Spearman’s rank correlation test between the relative abundance of OTUs in the milk or vagina and those in piglets were performed as well. Prevalent OTUs (> 20% in either the source or sink samples) were identified as potential transmitted OTUs if both OTU sharing permutation test, and Fisher’s exact test were significant or if Spearman’s rank correlation test was significant (*P* < 0.05) for an OTU present in all individuals. The phylogenetic tree of transmitted OTUs was constructed with RAxML [93]. Correlations between the intestinal gene expression levels and relative abundance of OTUs were tested with Spearman correlation [94, 95]. SPIEC-EASI [96] was applied for the inference of microbial ecological networks among the OTUs that were significantly associated with gene expression. The networks were visualized using Gephi [97].

## Supplementary information

**Additional file 1: Figure S1.** Experimental design and workflow of sample collection. Each tick denotes a time-point of sample collection.

**Additional file 2: Figure S2.** Rarefaction curves based on the Chao1 and Shannon index at increasing sequencing depth of intestinal mucosal, maternal and environmental samples.

**Additional file 3: Figure S3.** (A) Negligible influence of breed on the mucosa-associated microbiota at birth. Average weighted UniFrac, unweighted UniFrac and Bray-Curtis distance between individuals at birth within and between Rongchang and Landrace piglets. (B) Shift of α diversity of mucosa-associated microbiota with age across four intestinal segments based on Chao1 index (Values are Means ± SE; significance between groups was determined by Kruskal-Wallis test).

**Additional file 4: Figure S4.** PCoA of phylogenetic community composition based on unweighted UniFrac distance (A), and taxonomic community composition based on Bray–Curtis (B).

**Additional file 5: Figure S5.** β-diversity of the mucosa-associated microbiota, milk, skin, vagina and feces of sows, and environmental microbiota. PCoA of phylogenetic community composition based on weighted UniFrac (A), and taxonomic community composition based on Bray–Curtis (B).

**Additional file 6: Figure S6.** Distance comparison of microbial communities between mucosa-associated intestinal microbiota with that of maternal milk and feces. Values are Means ± SE; significance between intestinal segments was determined by pairwise Kruskal-Wallis test; * *P* < 0.05; ** *P* < 0.01; *** *P* < 0.001.

**Additional file 7: Figure S7.** Distance comparison of microbial communities between true compared with random sow-piglet pairs for fecal, vaginal and environmental microbiota (Values are Means ± SE; significance between intestinal segments was determined by pairwise Kruskal-Wallis test; * *P* < 0.05; ** *P* < 0.01; *** *P* < 0.001).

**Additional file 8: Table S1.** Primers used in the study.

**Additional file 9: Table S2.** Shift of PICRUSt-predicted KEGG pathways of mucosa-associated microbiota in small intestine and large intestine (*P* values by Welch’s t-test with a Benjamini–Hochberg FDR correction were computed by STAMP).

**Additional file 10: Table S3.** Different PICRUSt-predicted KEGG pathways of mucosa-associated microbiota between two clusters, small intestinal cluster (including the large intestinal samples at birth) and large intestinal cluster (except the large intestinal samples at birth). *P* values by Welch’s t-test with a Benjamini–Hochberg FDR correction were computed by STAMP.

**Additional file 11: Table S4.** Candidate transmitted OTUs identified from mother and environment.

**Additional file 12: Table S5.** Relative expression of intestinal function-related genes using the whole statistical model.

**Additional file 13: Table S6.** Contrasts for MUC1, MUC2 and pBD1 expression analyzed within each intestinal segment.

**Additional file 14: Table S7.** Correlation of the relative abundance of OTUs and expression of genes involved in the development of intestinal barrier function.

## Data Availability

The data generated or analyzed during this study are included in this published article, its supplementary information files, and publicly available repositories. Raw 16S rRNA gene sequences and study metadata were deposited in the National Center for Biotechnology Information—NCBI repository (BioProject accession number: PRJNA524979 [98] and PRJNA480348 [99]).

## Abbreviations

GI: Gastrointestinal
SFB: Segmented filamentous bacteria
PERMANOVA: Permutational multivariate analysis of variance
PCoA: Principal coordinates analysis
PBD: Porcine β-defensin
RegIII: Regenerative III
MUC: Mucin
AHR: Aryl hydrocarbon receptor
TLR4: Toll-like receptor 4
IL: Interleukin
