# Supplementary material for: Maternal milk and fecal microbes guide the spatiotemporal development of mucosa-associated microbiota and barrier function in the porcine neonatal gut
Source: BMC Biol. 2019 Dec 18;17:106. doi: 10.1186/s12915-019-0729-2 (PMC6921401; doi:10.1186/s12915-019-0729-2)
Supplement: Supplementary file 1 — Additional file 1: Figure S1. Experimental design and workflow of sample collection. Each tick denotes a time-point of sample collection. [file 12915_2019_729_MOESM1_ESM.pdf]

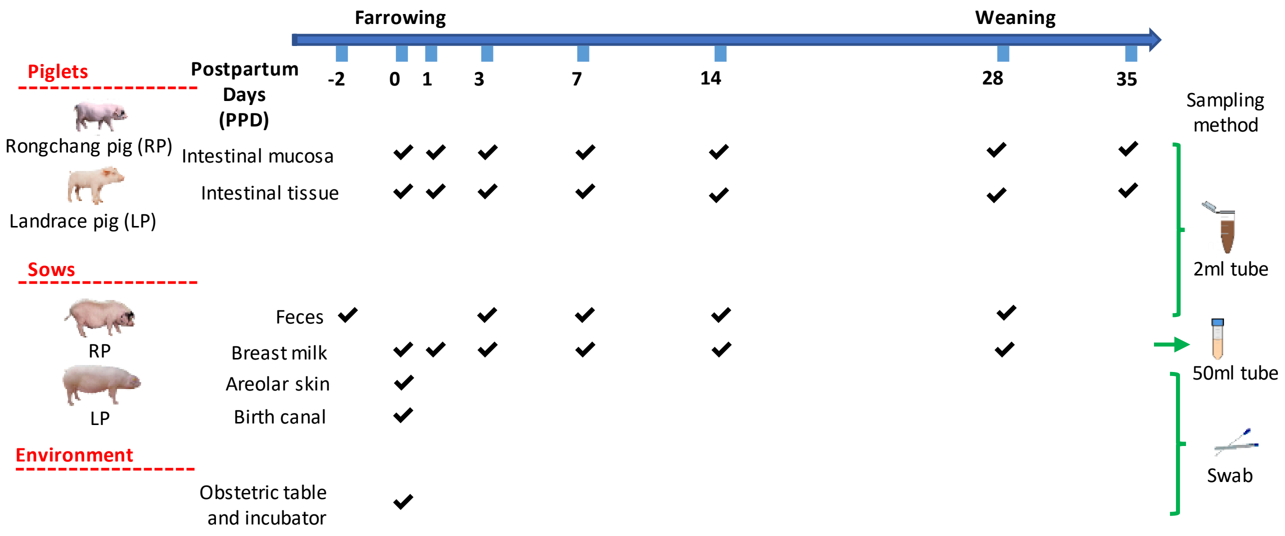
Farrowing
Weaning
Piglets
Postpartum Days (PPD)
-2
0
1
3
7
14
28
35
Sampling method
Rongchang pig (RP)
Intestinal mucosa
Intestinal tissue
Landrace pig (LP)
2ml tube
Sows
Feces
RP
Breast milk
50ml tube
Areolar skin
LP
Birth canal
Environment
Swab
Obstetric table and incubator
