# Supplementary figures and images for: Maternal milk and fecal microbes guide the spatiotemporal development of mucosa-associated microbiota and barrier function in the porcine neonatal gut
Source: BMC Biol. 2019 Dec 18;17:106. doi: 10.1186/s12915-019-0729-2 (PMC6921401; doi:10.1186/s12915-019-0729-2)

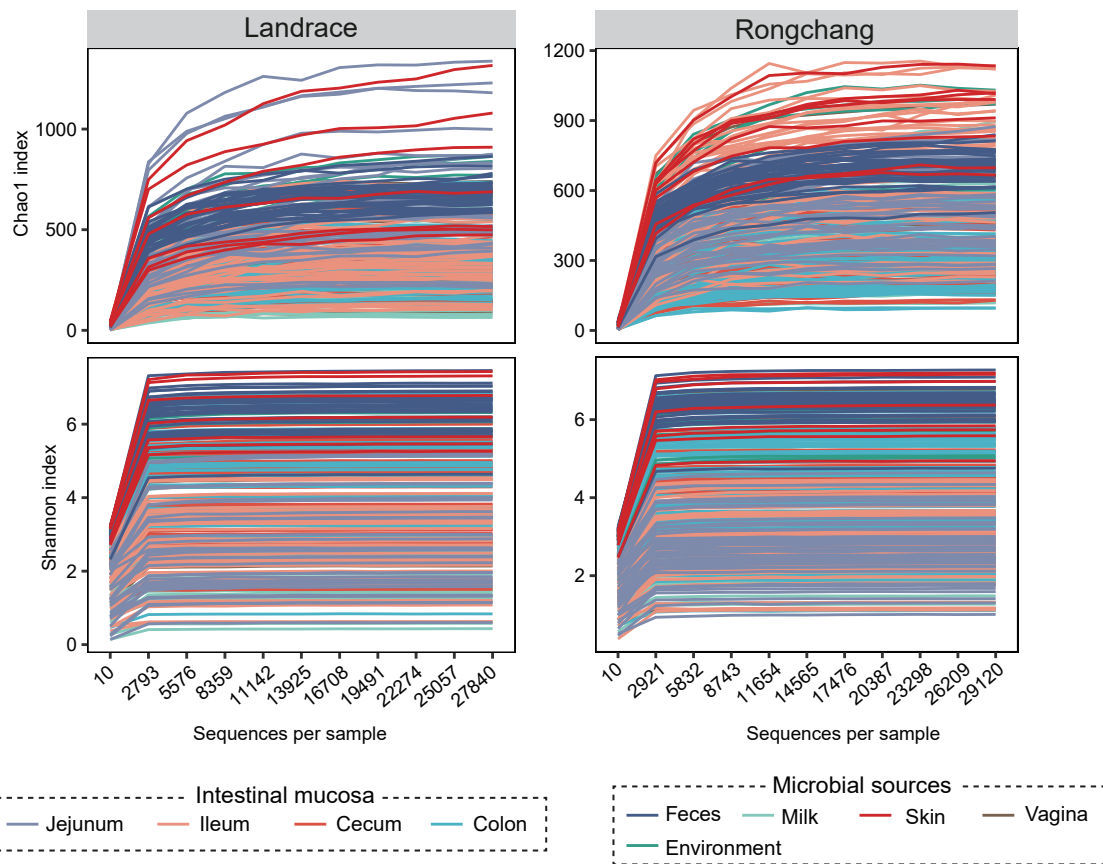

Supplement: Supplementary file 2 — Additional file 2: Figure S2. Rarefaction curves based on the Chao1 and Shannon index at increasing sequencing depth of intestinal mucosal, maternal and environmental samples. [file 12915_2019_729_MOESM2_ESM.pdf]

A.

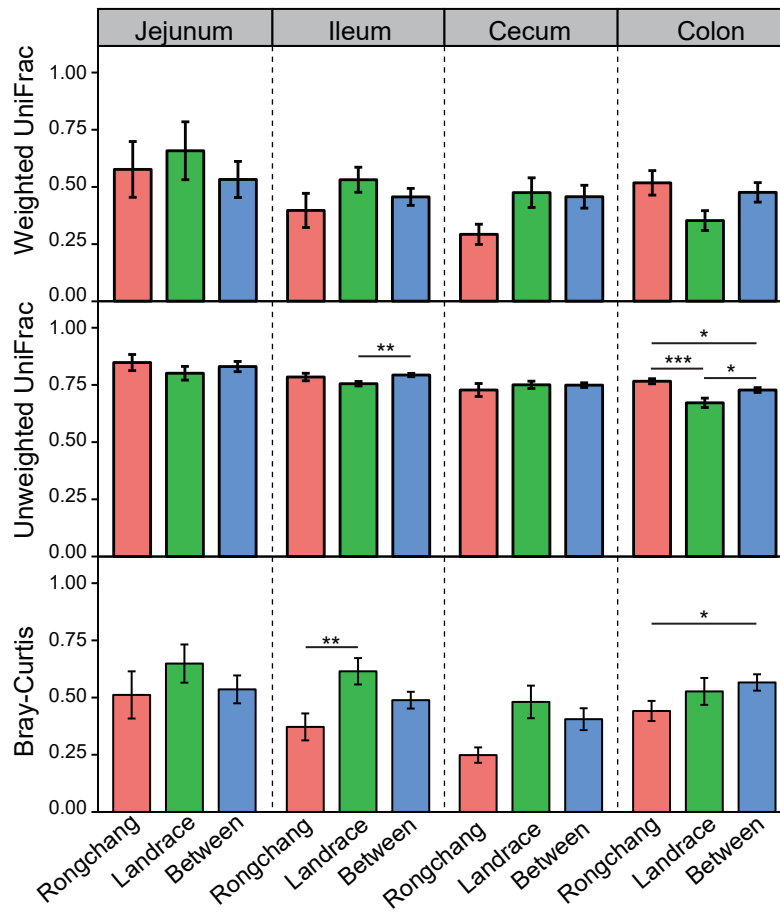

B.

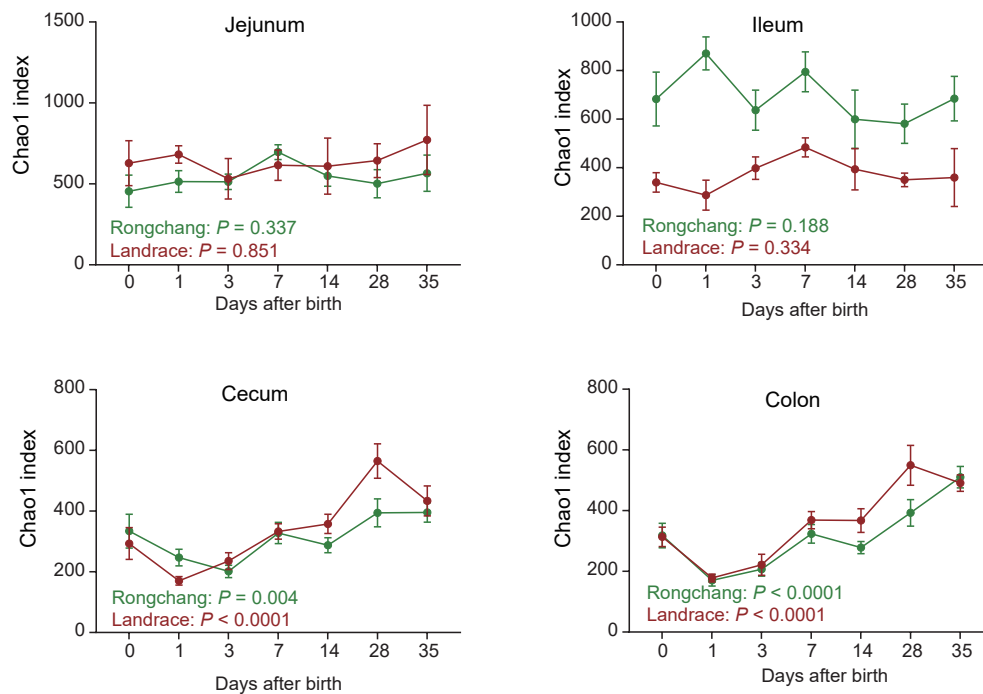

Supplement: Supplementary file 3 — Additional file 3: Figure S3. (A) Negligible influence of breed on the mucosa-associated microbiota at birth. Average weighted UniFrac, unweighted UniFrac and Bray-Curtis distance between individuals at birth within and between Rongchang and Landrace piglets. (B) Shift of α diversity of mucosa-associated microbiota with age across four intestinal segments based on Chao1 index (Values are Means ± SE; significance between groups was determined by Kruskal-Wallis test). [file 12915_2019_729_MOESM3_ESM.pdf]

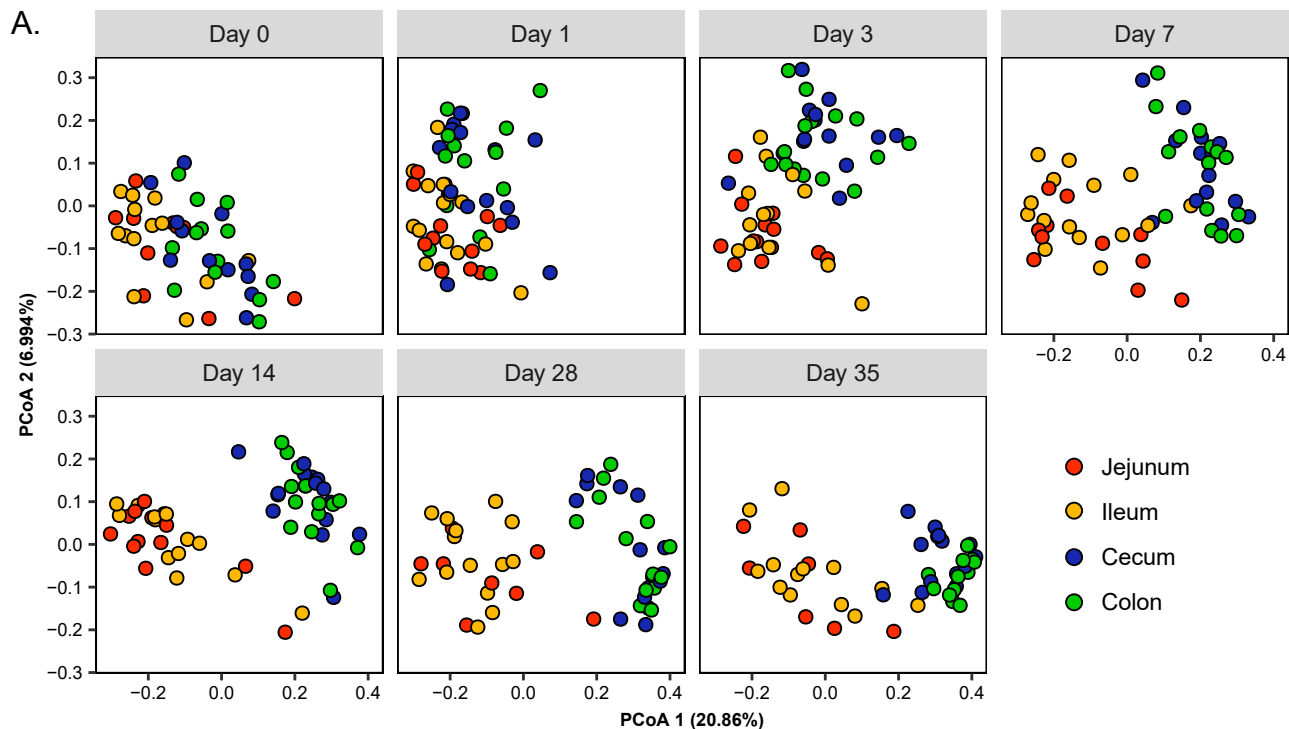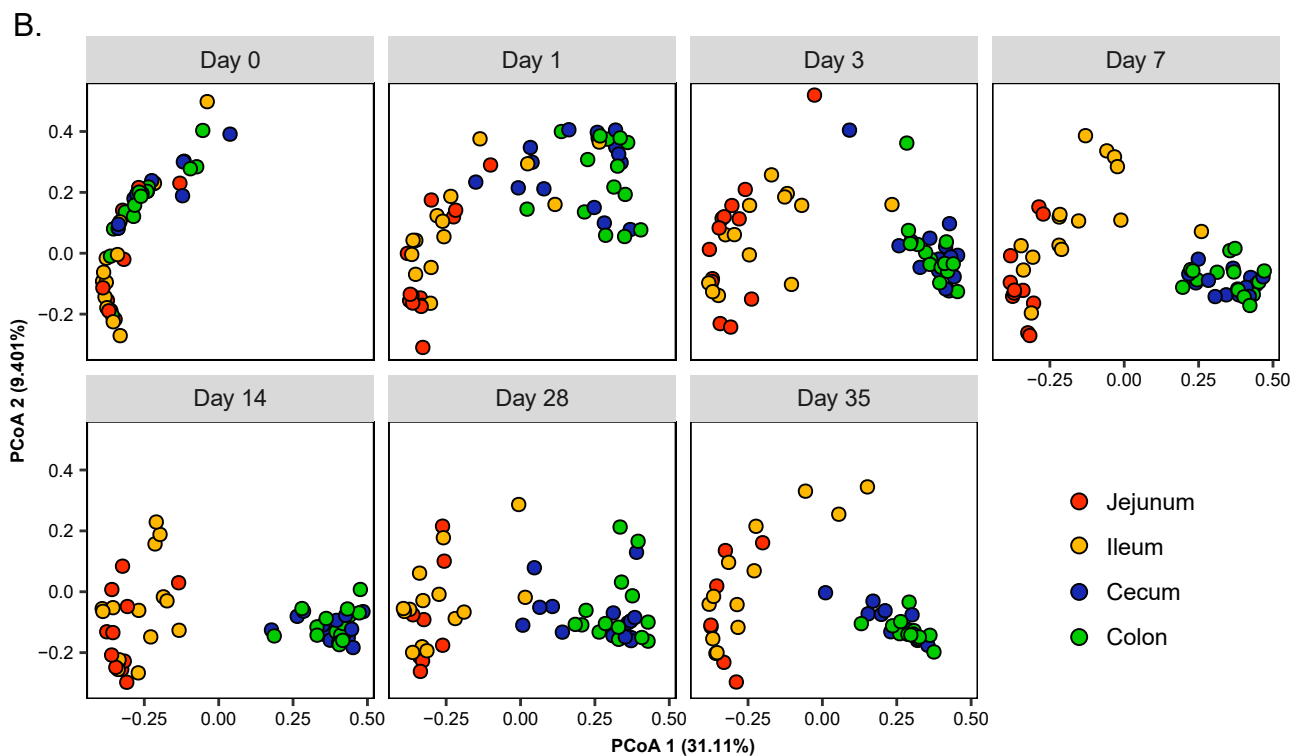

Supplement: Supplementary file 4 — Additional file 4: Figure S4. PCoA of phylogenetic community composition based on unweighted UniFrac distance (A), and taxonomic community composition based on Bray–Curtis (B). [file 12915_2019_729_MOESM4_ESM.pdf]

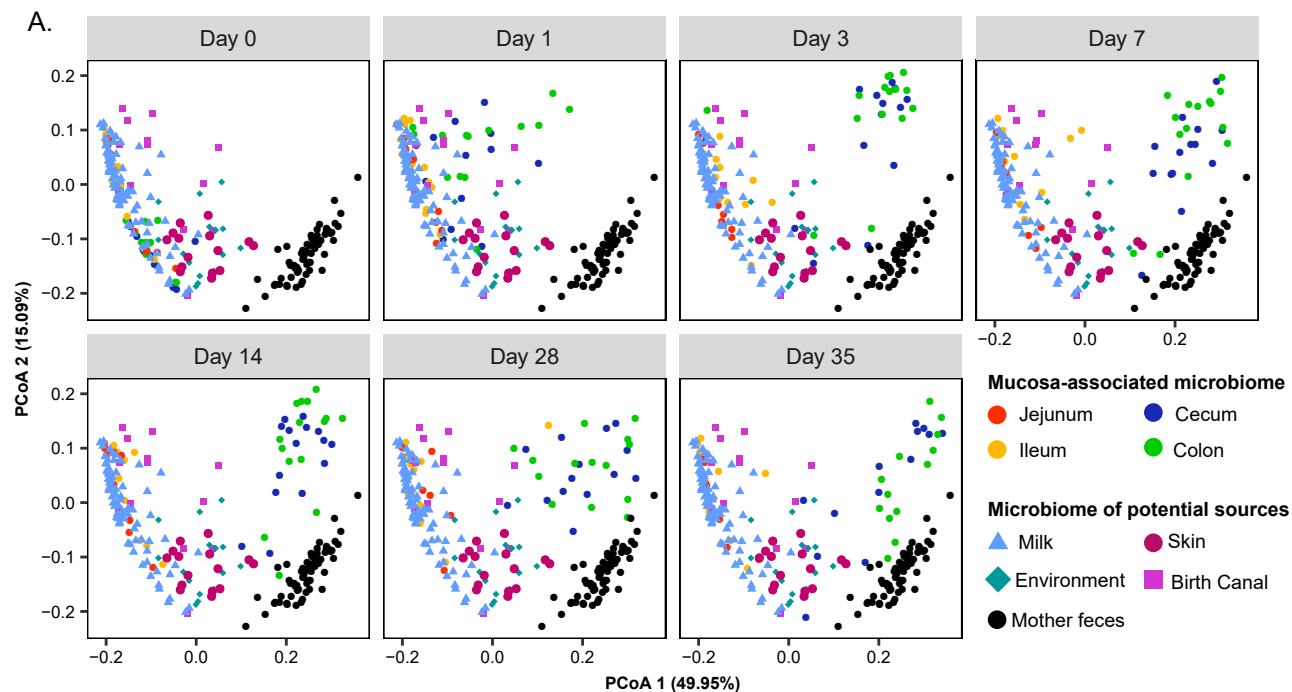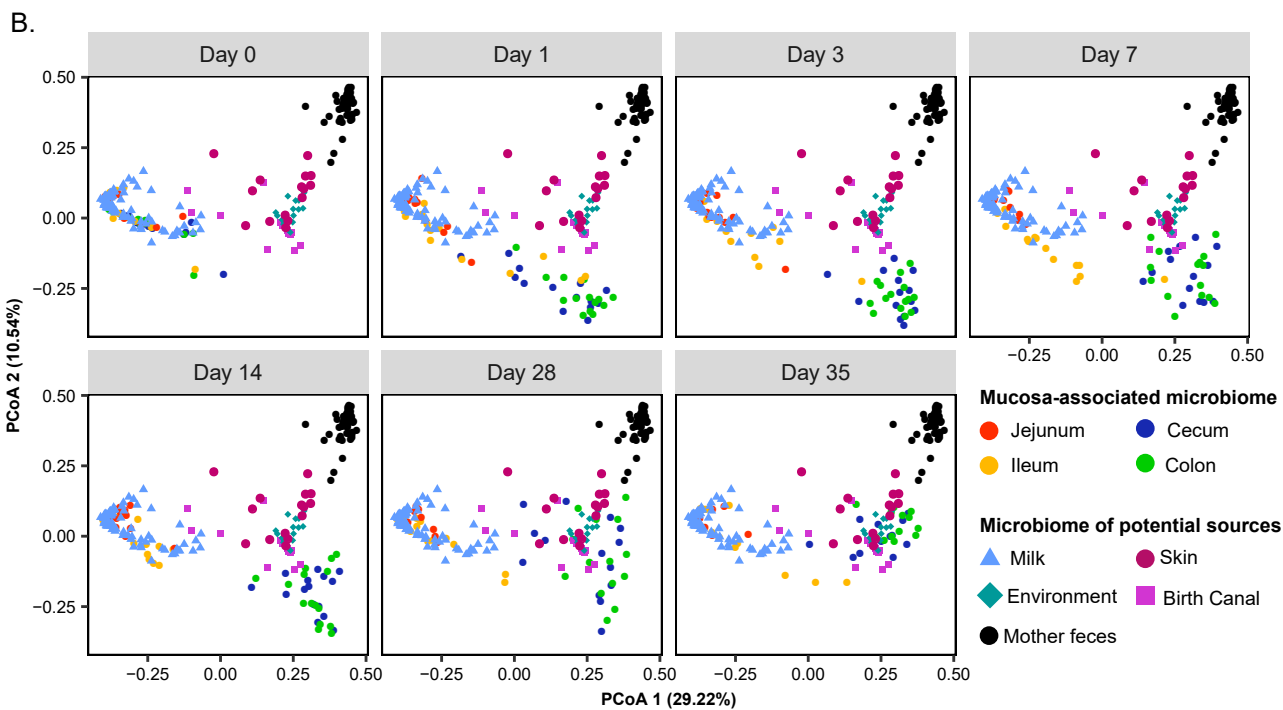

Supplement: Supplementary file 5 — Additional file 5: Figure S5. β-diversity of the mucosa-associated microbiota, milk, skin, vagina and feces of sows, and environmental microbiota. PCoA of phylogenetic community composition based on weighted UniFrac (A), and taxonomic community composition based on Bray–Curtis (B). [file 12915_2019_729_MOESM5_ESM.pdf]

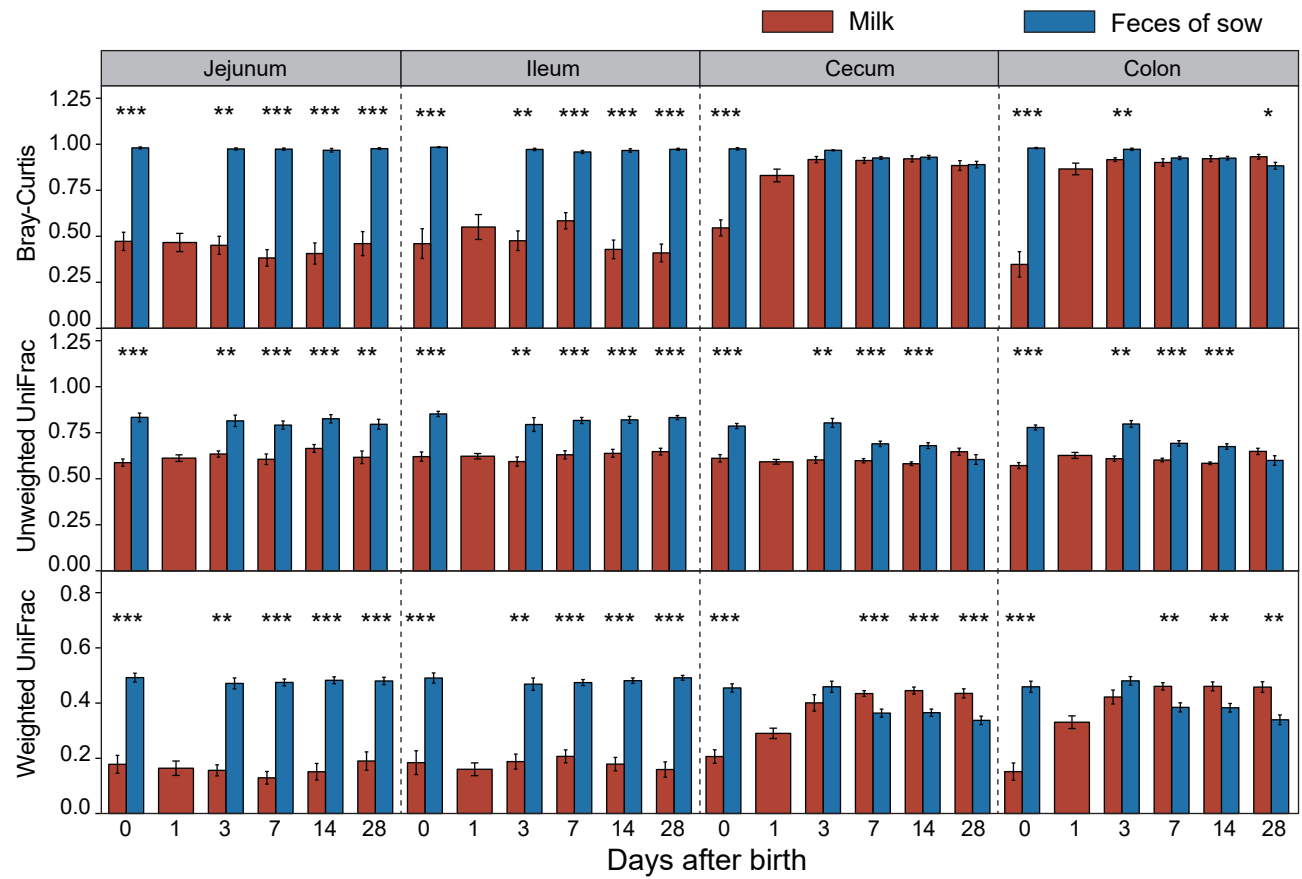

Supplement: Supplementary file 6 — Additional file 6: Figure S6. Distance comparison of microbial communities between mucosa-associated intestinal microbiota with that of maternal milk and feces. Values are Means ± SE; significance between intestinal segments was determined by pairwise Kruskal-Wallis test; * P < 0.05; ** P < 0.01; *** P < 0.001. [file 12915_2019_729_MOESM6_ESM.pdf]

Sow-piglet dyads

Random pair permutations

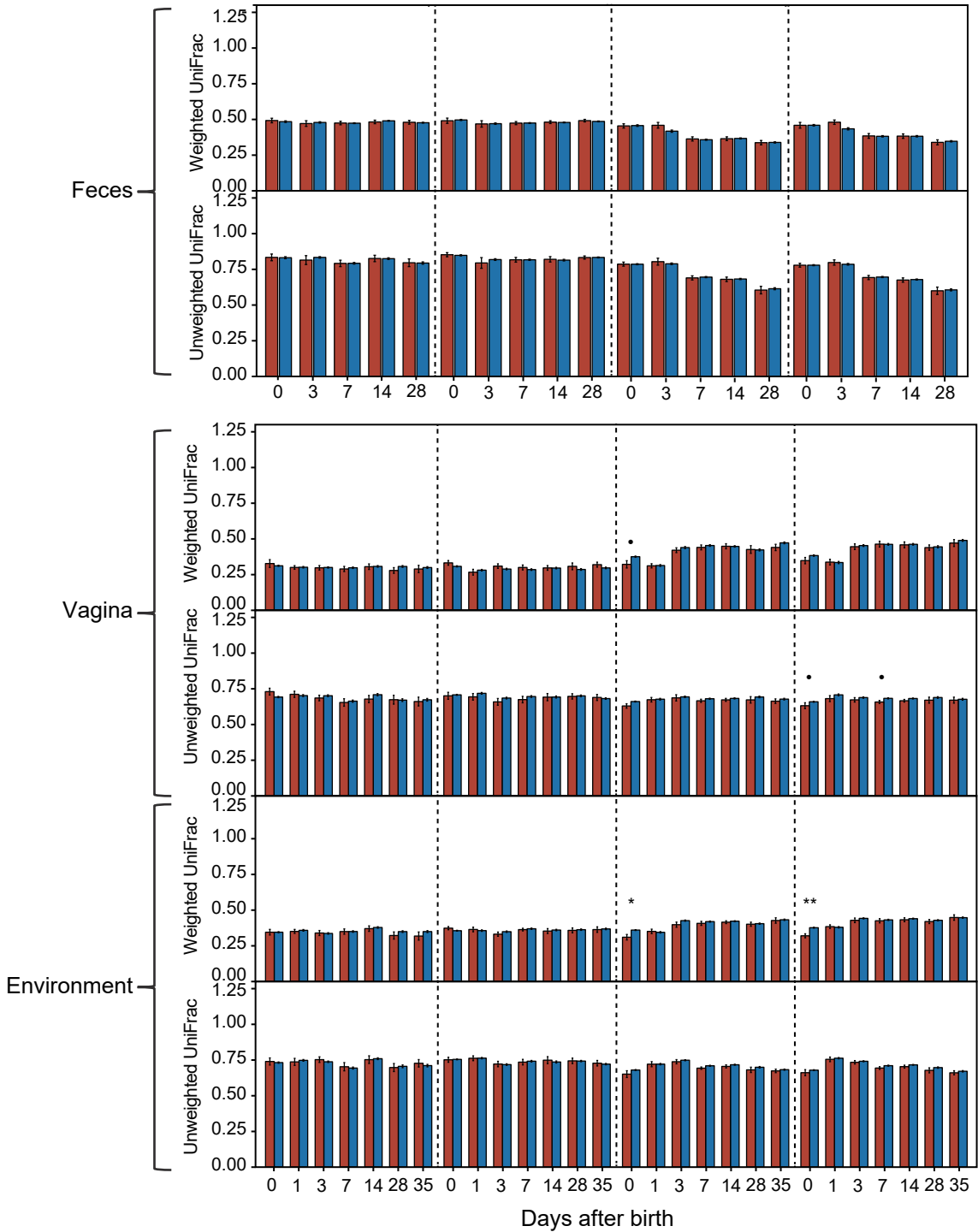

Supplement: Supplementary file 7 — Additional file 7: Figure S7. Distance comparison of microbial communities between true compared with random sow-piglet pairs for fecal, vaginal and environmental microbiota (Values are Means ± SE; significance between intestinal segments was determined by pairwise Kruskal-Wallis test; * P < 0.05; ** P < 0.01; *** P < 0.001). [file 12915_2019_729_MOESM7_ESM.pdf]
